# Influence of Ultrapulsed CO_2_ Laser, before Application of Different Types of Fluoride, on the Increase of Microhardness of Enamel* In Vitro*

**DOI:** 10.1155/2018/5852948

**Published:** 2018-08-06

**Authors:** Marcia Regina Cabral Oliveira, Pedro Henrique Cabral Oliveira, Luiz Henrique Cabral Oliveira, Ravana Angelini Sfalcin, Renato Araujo Prates, Ricardo Scarparo Navarro, Paulo Francisco Cesar, Alessandro Melo Deana, Maria Cristina Chavantes, Sandra Kalil Bussadori, Anna Carolina Ratto Tempestini Horliana

**Affiliations:** ^1^Postgraduate Program in Biophotonics Applied to Health Sciences, Universidade Nove de Julho, UNINOVE, São Paulo, Brazil; ^2^Undergraduate Student, Postgraduate Program in Biophotonics Applied to Health Sciences, Universidade Nove de Julho, UNINOVE, São Paulo, Brazil; ^3^Postgraduate Program in Bioengineering and Biomedical Engineering, Universidade Brazil, São Paulo, Brazil; ^4^Department of Biomaterials and Oral Biology, University of São Paulo, São Paulo, Brazil; ^5^Postgraduate Program in Medicine and Biophotonics, Universidade Nove de Julho, UNINOVE, São Paulo, Brazil; ^6^Postgraduate Program in Rehabilitation Sciences, Universidade Nove de Julho, UNINOVE, São Paulo, Brazil

## Abstract

**Objective:**

To evaluate the influence of ultrapulsed CO_2_ laser in combination with commercial fluoride products in order to verify the increase of microhardness of artificial enamel caries lesions.

**Materials and Methods:**

Bovine enamel specimens were prepared, and artificial enamel caries lesions were created. Teeth were randomly divided into 5 groups (n=10): treated with laser (L), laser + neutral fluoride gel 2% (LNF), laser + acidulated phosphate fluoride gel 1.23% (LAFG), laser + acidulated fluoride mousse 1.23% (LAFM), and laser + fluoride varnish 5% (LFV). Microhardness was evaluated at baseline, after caries induction, after CO_2_ laser irradiation + fluoride treatment in the 1st week, and after fluoride treatment at 3rd and 5th week.

**Results:**

There was a decrease in microhardness in all groups after artificial enamel caries lesion formation; no increase in microhardness was found in the first and third weeks in all groups (p > 0.05). In the fifth week, an increase in microhardness occurred in all groups (p < 0.05).

**Conclusion:**

Although CO2 laser irradiation in combination with different commercial fluoride products was capable of increasing microhardness on enamel caries lesions in bovine tooth enamel it is necessary to confirm these results by testing the isolated effect of fluoride on enamel surface microhardness. Also, although microhardness was higher in the fluoride varnish group than in the other groups in the fifth week it is not possible to discard the best effect of fluoride varnish treatment on absence of artifacts that may occur with the other fluoride treatments.

**Clinical Relevance:**

In order to prove that CO2 laser may contribute to an increase in microhardness when applied to enamel lesions in combination with different commercial fluoride products it is necessary to conduct additional studies. Also, higher microhardness of fluoride varnish group should be carefully considered.

## 1. Introduction

Dental caries is still a public health problem, and untreated caries lesions are the most prevalent chronic oral problem, as observed in the Global Burden of Disease study in 2010 [1, 2]. Approximately 35% of the world population is affected by this condition, which exerts a considerable influence on quality of life and has implications with regard to systemic health [3–5]. Early enamel caries lesions are formed during alternating periods of demineralization and remineralization [6]. The net gain or loss in minerals from the enamel structure determines whether tooth decay will advance, stabilize, or regress. Minimally invasive approaches have been the treatment of choice in order to manage caries lesions with thoroughgoing preservation of tooth structures [7], i.e., the application of different types of fluoride agents, in different concentrations. It has been reported that long-term exposure to fluoride with levels of 0.095-0.190 ppm fluoride in the saliva may be sufficient to arrest caries progression [8]. Fluoride acts to prevent caries by being a biocide [9, 10] and by reducing the solubility of enamel and dentin through its incorporation into tooth tissue to form fluoroapatite [11–13]. Also, fluoride acts to remineralize damaged tooth tissue following demineralization [11, 13, 14]. It has been demonstrated that a reaction occurs between the soluble fluoride found in commercial products and the tooth's minerals thus resulting in the precipitation of CaF_2_ mineral-like deposits in the biofilm [15]. This loosely bound fluoride layer formed on both the enamel surface and in plaque during the demineralization process in clinical situations can stabilize the mineral apatite and remineralize the enamel, making it substantially more resistant to demineralization [16–18]. However, successful treatment requires the early detection of caries, adequate hygiene technique, and the use of fluoride agents [19, 20]. Studies have demonstrated the effectiveness of fluoride in the prevention of caries, but data on the most appropriate vehicles for all situations have been studied until now [5, 17, 21–23]. Researchers have been applying different minimally invasive treatments to treat existing, noncavitated, early caries lesions (e.g., white spots) using different technologies [24–29].

The use of fractionated CO_2_ laser before and after the application of fluoride was investigated [16]. When it was used before fluoride therapy, the enamel caries lesions were remineralized. Some authors [29] evaluated the use of acidulated phosphate fluoride combined with CO_2_ laser (*λ* = 10,600 nm) with 11.3 or 20 J/cm^2^ and 0.4 or 0.7 W and concluded that the irradiation significantly enhanced CaF_2_ uptake by demineralizing the enamel and inhibiting lesion progression. In view of these promising results it is necessary to test whether the association of laser with other commercial forms of fluoride would also increase enamel microhardness [30, 31].

Thus, the aim of this study was to evaluate if the association of ultrapulsed CO_2_ laser with commercial fluoride products increases microhardness of artificial enamel caries lesions.

## 2. Material and Methods

Brazilian law 11/797/08 published on October 8th, 2008 (article 3, paragraph 3), defines animal experimentation as procedures conducted with live animals. Since all tooth samples were obtained* postmortem* from discards of animals raised for commercial slaughter, this study did not require the approval of an animal research ethics committee.

### 2.1. Preparation of Enamel Specimens and Artificial Caries-Like Lesion Formation

**Figure 1:**
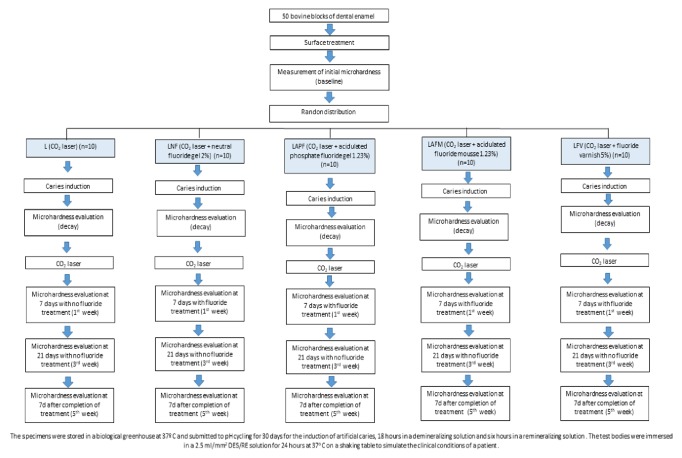
Flow chart.

Fifty extracted bovine teeth were cleaned and kept in 0.1% thymol solution at 4°C. The vestibular surface of the teeth was abraded with silicon carbide sandpaper in water (grits 400, 600, and 1200) (Buehler) and polished with a felt disc and diamond pastes with granulations of 0.5, 1, 2, and 3 *μ*m. Enamel blocks (7 X 4 X 4 mm) were cut from the prepared vestibular surface, using double-sided diamond discs and a low-speed drill with cooling (Isomet 1000, Buehler, Lake Buff, IL, USA). Blocks were covered with two layers of acid-resistant nail varnish (Colorama®, São Paulo, Brazil), except for the flattened exposed enamel surface [27, 31]. The specimens were kept in a stove at 37°C and underwent the first round of pH cycling for 30 days for demineralization (2.5 ml solution/mm^2^ of enamel surface) [32, 33]. The fifty prepared specimens were randomly distributed into five groups (n= 10) (Figure 1).

### 2.2. Laser Irradiation

After artificial enamel caries lesion formation, CO_2_ laser irradiation was applied to the enamel blocks, one time only, before the application of different types of fluoride treatments.

### 2.3. Laser Parameters

Ultrapulsed CO_2_ laser (*λ* = 10,600 nm) (Ultralase 30, South, Atl, USA) was used in pulsed mode, with an average radiant power of 48 mW, peak power of 1W, aperture diameter of 0.20 cm, power density at aperture of 1.20 W/cm^2^, beam spot size at target of 0.04 cm^2^, irradiance at target of 1.20 W/cm^2^, duty cycle of 5%, duty exposure duration of 0.715 seconds, exposure duration of 14.3 s, pulse duration of 0.005 s, pulse-off time of 0.099 s, pulse repetition frequency of 9.6Hz, number of pulses at irradiated area 176, radiant exposure of 0.858J/cm^2^, radiant energy of 34 mJ, energy per pulse of 5 mJ, irradiated area of 0.04cm^2^, number and frequency of treatments being one. With a reduced duty cycle (5%) it was possible to deliver a large amount of radiant energy with the laser CO2 off for a long interval.

### 2.4. Fluoride Treatment and pH Cycling

After CO_2_ laser irradiation, experimental groups G2-LNF, G3-LAFG, G4-LAFM, and G5-LFV were submitted to topical fluoride applications once a week, following the manufacturers' recommendations for application (Table 1), for a total period of 5 weeks. During the topical fluoride treatments, both aforementioned experimental groups and the control group (G1-L, without fluoride) were submitted to the same pH cycling treatment as applied before laser irradiation. However, the periods of immersion in DE-RE solutions were changed. The pH cycling model was as follows: Teeth were put for six hours in DE solution, so they were rinsed with distilled water and dried with absorbent paper. Thus, they were put for 18 hours in the RE solution at 37°C with agitation (MacLab, Jacareí, SP) [23, 31, 33]. All excess fluoride product was immediately removed with absorbent paper and the specimens were immediately placed back into pH cycling.

In the varnish group, we applied it. Then, we waited for 1 min for solvent evaporation and then the excess was removed with the absorbent paper. Immediately the specimen was placed in RE solution, and the tubes were kept under stirring throughout the assay, to simulate clinical conditions. After creating the early enamel caries lesion, samples were immersed for 18 hours in the remineralizing solution and for 6 hours in the demineralizing solution to simulate an oral environment. This second pH cycling was carried out along with the fluoride treatments.

### 2.5. Surface Microhardness Evaluation

Surface microhardness was determined by a Knoop indenter with a load of 50 g for 20 seconds using HMV Micro Hardness Tester (Shimadzu, Japan). Five indentations were made at a distance of 100 *μ*m from each other and the mean was used for the analysis [33]. Surface microhardness was determined on five occasions: (1) on sound enamel (baseline); (2) immediately after enamel caries lesion formation; (3) seven days (1 week) after the beginning of the treatments; (4) after 21 days (3 weeks); and (5) 35 days (1 weeks) after the completion of treatment (the total duration of treatment was 4 weeks). Only one operator performed all the microhardness assays. The samples treatments were also performed by only one operator who was blinded to the results of the microhardness assays.

### 2.6. Statistical Analysis

Mean and standard error of the mean (SEM) values were calculated for all groups and compared among the readings taken at baseline, after enamel caries lesion formation, and one week and three weeks after enamel caries lesion formation. Microhardness was also evaluated one week after the fourth-week treatment (after the completion of treatment). The groups were compared at each evaluation time (intergroup analysis) as well as individually over time (intragroup analysis). The Shapiro-Wilks test demonstrated that the data exhibited normal distribution. Two-way repeated measure analysis of variance (ANOVA) was used to make the comparisons, followed by Bonferroni's post hoc test. The level of significance was *α* = 0.05. All statistical analyses were performed using the Statistical Package for Social Sciences (SPSS).

## 3. Results

**Figure 2:**
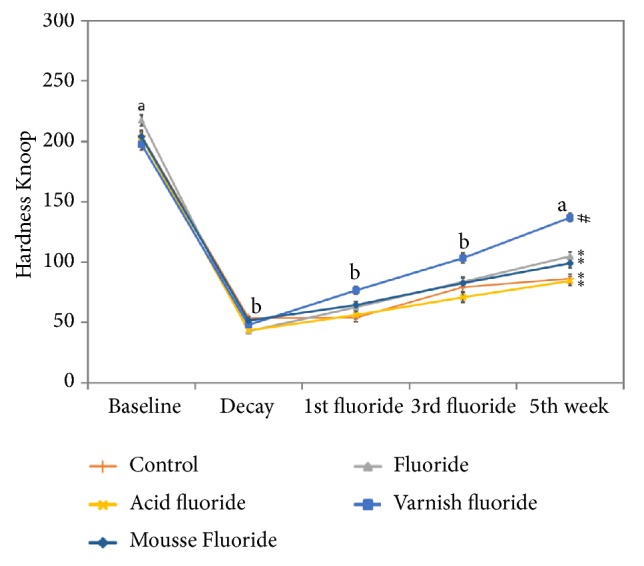
Evaluation of microhardness of bovine tooth enamel before and after enamel caries lesion induction and after the first, third, and fifth weeks. Different letters mean statistically significant different times. Different symbols mean statistically significant different treatments. Mean values (n=10) of surface microhardness of the tooth enamel according moment of analysis and treatment. Distinct letters (a, b) indicate statistical difference among the times of analysis. Different symbols (# *∗*) indicate statistical difference among the treatments. Vertical bars indicate standard error of the mean (Bonferroni test, p<0.05).

Intergroup (treatment analysis; p = 0.0230, two-way repeated measures ANOVA) and intragroup (time analysis; p < 0.0001, two-way repeated measures ANOVA) differences were found in the microhardness analysis (Figure 2). Although the intercept presented statistically significant differences (times*∗*treatment; p < 0.0001, two-way repeated measures ANOVA) this is likely due to the decrease in hardness (after the induction of decay) and its subsequent increase (due to treatment); thus such differences are expected. In the intragroup analysis, a significant reduction in microhardness was found after the induction of artificial enamel caries lesion (decay) in all groups (p < 0.05). From the first to the third week, no increase in microhardness values occurred in any of the groups (p > 0.05) in comparison to the decay period. In the fifth week, however, an increase in microhardness occurred in all groups in comparison to the decay period. In the intergroup comparisons, no differences were found between the baseline, decay, one-week, or three-week evaluations (p > 0.05). At the five-week evaluation, however, the fluoride varnish group exhibited significantly higher microhardness (p < 0.05) than the other groups.

## 4. Discussion

Products with a high fluoride concentration are indicated for individuals with high caries activity as well as special needs patients [14, 17, 18]. Fluoride gels, mousses, and varnishes have been employed in Pediatric, Preventive, and General Dentistry, but questions remain regarding application time and the capacity of such products to affect the remineralization of early caries lesions in dental enamel. In the present study, the increase in surface microhardness of enamel was followed for a four-week period, which is the treatment time established for early enamel caries lesions. An additional evaluation (5th week) was performed to verify microhardness of the enamel surface one week after completion of treatment. The surface microhardness values for the 5th week did not differ from those found at the baseline. This was because, while there was an increase in the surface microhardness during the 5 weeks, the samples were immersed in the demineralizing solution for 6 hours and in the remineralizing solution for 18 hours, for 5 weeks. Thus, there was a large period of immersion in the remineralizing solution which contains calcium and phosphate in the composition. Then, when associaded with fluoride applications, it is expected that an increase in mineral presence on the enamel surface in all groups occurs, also in control group.

A previous study showed the importance of a Ca prerinse prior to a F rinse as this process forms substantial amounts of CaF2-like deposits [15]. Studies have demonstrated greater efficiency in the remineralization process when acidulated phosphate fluoride gel is used [14, 16, 17, 34]. On the other hand, fluoride gel should be used with caution when treating patients in special needs groups and preschool children due to the risk of ingestion and gastric harm stemming from the toxicity [35]. Few studies propose the use of CO_2_ laser for the treatment of early enamel caries lesion due to the heterogeneity, porosity, and fragility of this tissue. As carious tissue is more porous and has different energy absorption characteristics, an inappropriate dose of energy could exert a negative influence on treatment [26]. The most likely hypothesis for the inhibiting effects of laser on caries is the fusion of hydroxyapatite crystals [26, 36]. It has been demonstrated that lasers alter the composition of water and carbonate of the enamel's superficial structure, thus reducing the acid reactivity of the mineral [37, 38]. The combination of CO_2_ laser with fluoride treatments could have a synergistic effect and be more efficient than either fluoride or CO_2_ laser irradiation alone [38]. Further studies should investigate Raman spectrum or Scanning Electron Microscopy (SEM).

In order to further confirm our hypothesis, it is necessary to confirm the results of this study by testing the isolated effect of fluoride on enamel surface microhardness. The greater hardness achieved with the varnish may be explained by the high concentration of fluoride and considerable substantivity of this product. Also, it is important to note that a limitation of this study is the use of only microhardness to evaluate the effect of the treatments as there exists the impact of artifacts provoked on the dental surface by acidic and highly concentrated fluoride products. As previously discussed [39, 40] loosely bound fluoride products are formed in enamel by partial dissolution of a layer of enamel and reprecipitation of fluoride salts. The loosely bound products formed on dental surfaces are subsequently dissolved in the saliva and this dissolution provokes changes in the dental surface. Thus, microhardness may not be the best choice to evaluate the effect of the acidulated sodium fluoride group (one of the groups used in this study, LAFG). Thus, the better performance of F-varnish is not a surprise, because it is a neutral product and most of the fluoride in it is insoluble by the organic solvent (ethanol) used to dissolve the varnish matrix. It is not possible to discard the best effect of F-varnish treatment on absence of artifacts that may occur with the other fluoride treatments.

Also, of note, the L group (control) presents levels of microhardness similar to that achieved in other groups with laser irradiation combined with fluoride products (LNF, LAFG, LAFM, and LFV groups). Further studies must be conducted to evaluate the microhardness of enamel caries lesions after CO_2_ laser application alone, without the following immersion of the specimens into a remineralizing solution, in order to confirm the findings of our study. Also, these results should be analyzed with caution because of the remineralizing potential of the DES/RE solution used in this study. Further studies are necessary to test the temperature achieved with ultrapulsed irradiation. This parameter was performed with considerable spacing to minimize the deposition of accumulated energy, which may have prevented an increase in the temperature of the pulp [17, 32].

Although CO2 laser irradiation in combination with different commercial fluoride products was capable of increasing microhardness on enamel caries lesions in bovine tooth enamel it is still necessary to confirm these results by testing the isolated effect of fluoride on enamel surface microhardness. Also, although microhardness was higher in the fluoride varnish group than in the other groups in the fifth week, it is not possible to discard the best effect of fluoride varnish treatment in the absence of artifacts that may have occurred with the other fluoride treatments. Further studies should investigate Raman spectrum or Scanning Electron Microscopy (SEM) of fluoride groups alone or associated with fluoride to understand the mechanism of action of this interaction. In conclusion, all the tested groups in this study showed increased microhardness of artificial enamel caries lesions.

## Figures and Tables

**Table 1:** Information of the materials.

**Commercial name**	**Composition**	**Fluoride Concentration**	**Manufacturer**	**Time of contact: product/enamel**
Fluoride Gel ®	Acidulated sodium fluoride	1.23%	Sultan Topex, DFL	1 min, after which it was removed with absorbent paper

Flugel ®	NaF	2%	Sultan Topex, DFL	1 min, after which it was removed with absorbent paper

Fluor Care®	Acidulated sodium fluoride	1.23%	FGM	1 min, after which it was removed with absorbent paper

Duraphat®	NaF	5%	Colgate	1 min, after which it was removed with absorbent paper

Demineralizing solution	H_2_O, KOH, CH _3_COOH, C_2_H3NaO_2_, H_3_PO_4_, Ca pH 3.5 to 4	0.030 ppm	Fórmula & Ação Pharmacy	pH cycling

Remineralizing solution (artificial saliva)	H_2_O, HCL, KOH, CaCl_2_, (HOCH_2_)_3_CNH_2_, Ca, P, Tris buffer pH 7	0.050 ppm	Fórmula & Ação Pharmacy	pH cycling

## Data Availability

The data used to support the findings of this study are included within the article.

## Supplementary Materials

Supplementary Materials Figure 1: electron micrograph of normal bovine dental enamel magnified 300 and 10000 x. Figure 2: electron micrograph of bovine dental enamel following induction of artificial caries magnified 300 and 10000 x.
