# Supplementary material for: Influence of Ultrapulsed CO2 Laser, before Application of Different Types of Fluoride, on the Increase of Microhardness of Enamel In Vitro
Source: Biomed Res Int. 2018 Aug 6;2018:5852948. doi: 10.1155/2018/5852948 (PMC6106794; doi:10.1155/2018/5852948)

Here we present an electron micrograph of sound and carious enamel surface as supplemental material, only to prove that the lesions retain a relatively well-preserved surface layer.

*Surface analysis*

Figure 1 displays the same region of normal bovine dental enamel magnified 300 and 10000 times. The topography of the enamel is uniform, with the absence of eroded areas as well as the presence of tiny cracks, due to the method employed. The larger magnification illustrates the natural surface arrangement.

**Figure 1** – Electron micrograph of normal bovine dental enamel magnified 300 and 10000 x

**
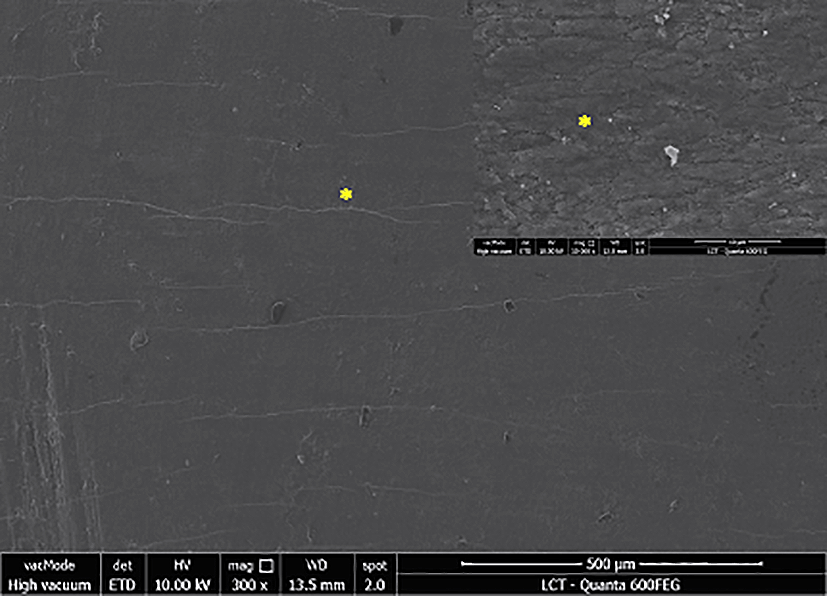
**

Figure 2 displays bovine dental enamel following the induction of artificial caries. The lower magnification shows craters stemming from the mineral loss caused by caries. These pores characterize the effective creation of a white spot lesion *in vitro*.

**Figure 2** – Electron micrograph of bovine dental enamel following induction of artificial caries magnified 300 and 10000 x


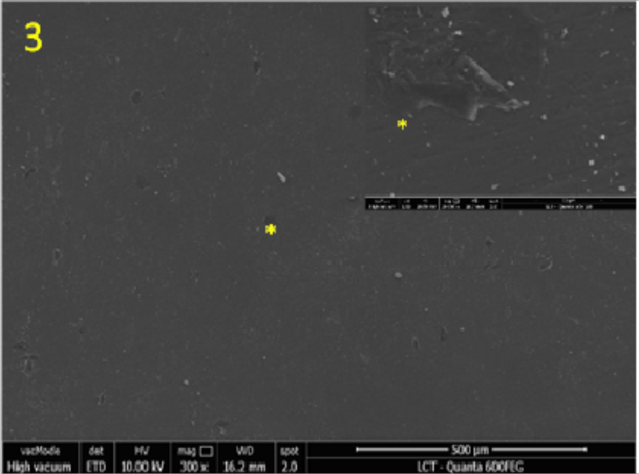

Supplement: Supplementary Materials — Figure 1: electron micrograph of normal bovine dental enamel magnified 300 and 10000 x. Figure 2: electron micrograph of bovine dental enamel following induction of artificial caries magnified 300 and 10000 x. [file 5852948.f1.docx]
